# Supplementary material for: Resolution limit of the eye — how many pixels can we see?
Source: Nat Commun. 2025 Oct 27;16:9086. doi: 10.1038/s41467-025-64679-2 (PMC12559231; doi:10.1038/s41467-025-64679-2)
Supplement: Supplementary file 1 — Supplementary Information [file 41467_2025_64679_MOESM1_ESM.pdf]

Supplementary Information

Contents

1 Historical context of visual acuity standards 1
2 Visual acuity units conversion 1
2.1 Snellen Fraction . . . 1
2.2 logMAR . . . 2
2.3 Pixels-per-degree . . . 2
3 Methods 2
3.1 Apparatus . . . 2
3.2 Stimuli . . . 4
3.3 Prior psychometric function estimation . . . 5
3.4 Psychophysical method . . . 5
3.5 Data analysis . . . 6
4 Results and discussion 6
4.1 Individual observer data . . . 6
4.2 Relationship with visual acuity . . . 7
4.3 Parameters of the resolution limit model . . . 8
4.4 Probability distribution across the population . . . 10
4.5 The effect of viewing distance . . . 10
4.6 Foveated filtering . . . 11
References 13

1 Historical context of visual acuity standards

As explained by Velasco e Cruz (1990)[1], Snellen's widely used visual acuity chart was based on Helmholtz's (1962)[2] report of the resolving power of the human eye, which was 1 minute of arc. However, this value actually represented twice the width of the wire grating used in Helmholtz's experiments, corresponding to a full square grating cycle. For the Snellen chart design, approximately 0.5 minutes of arc should have been used. The Snellen chart is designed such that the letters on the 20/20 line subtend an angle of 5 arc minutes, with each distinguishing feature subtending 1 arc minute of visual angle when viewed at 20 feet or 6 meters[3,4]. This has led to the historical misconception that 1 arc minute, or 60 ppd, is the critical resolution of the human eye and is often wrongly considered sufficient for display purposes.

2 Visual acuity units conversion

Visual acuity of angular resolution of the human eye is represented in many different units in the literature. Understanding these various units and how to convert between them is necessary for interpreting results and comparing data across different studies and contexts. The primary units of visual acuity include Snellen fraction, pixels per degree (ppd), and logMAR. Below are the mathematical formulae for conversion between these units, along with explanations.

2.1 Snellen Fraction

The Snellen fraction is one of the most common ways to express visual acuity. It is represented as a fraction, where the numerator indicates the testing distance (usually 20 feet or 6 meters), and the denominator indicates the distance at which a person with normal vision can read the same line of the eye chart. In simple terms, it is a ratio representing the visual acuity of an individual compared to normal vision.

$$r_{\text{snellen}} = \frac{d_{\text{chart}}}{d_{\text{norm}}}, \tag{A}$$

where, r_{snellen} is the Snellen fraction, d_{chart} is the distance between the observer and the chart, and d_{norm} is the distance at which a normal eye can read the line. For example, a Snellen fraction of 20/40 means that the test subject can read at 20 feet what a person with normal vision can read at 40 feet.

2.2 logMAR

LogMAR (logarithm of the Minimum Angle of Resolution) is a measure of visual acuity that provides a more intuitive scale. The logarithmic scale provides a more linear representation of visual acuity and is widely used in research and clinical settings. It is the minimum angle in minutes that can be resolved by an observer. The value is related to Snellen fraction as follows:

$$\log \mathrm{MAR} = \log_{10} \frac{1}{r_{\mathrm{snellen}}}. \tag{B}$$

For example, if the Snellen fraction is 20/40, then:

$$\log \mathrm{MAR} = \log_{10} \frac{40}{20} = \log_{10}(2) = 0.3010. \tag{C}$$

To convert from logMAR to Snellen fraction:

$$r_{\mathrm{snellen}} = \frac{1}{10^{\log_{10} \mathrm{MAR}}}. \tag{D}$$

2.3 Pixels-per-degree

Pixels per degree (ppd) is a measure of spatial resolution used in digital displays, indicating how many pixels occupy one degree of visual angle. The ppd can vary across the display due to differences in viewing angle at different points on the screen, particularly at the edges compared to the centre[5]. However, for practical purposes and consistency across studies, we use the ppd formula for the centre of the screen:

$$\mathrm{ppd} \approx \frac{\pi}{360 \cdot \arctan \frac{0.5 d_{\mathrm{width}}}{r_{\mathrm{w}} d_{\mathrm{v}}}}, \tag{E}$$

where d_{width} is the width of the screen, r_{w} is the display pixel resolution along the width, and d_{v} is the viewing distance between the observer and the display. In our experiments, the stimulus was always located at the centre of the screen, which was far enough for the approximation to hold. The eccentricity measurements used the distance to the horopter, which ensured correct calculations.

In the context of resolving power, the pixel-per-degree value relates to the viewer's visual acuity limit. For example, 20/20 vision is equivalent to a minimum resolving angle of 1 arc minute. For displays, this would mean that a pixel, or the smallest resolvable element, should span 1/60th of a visual degree or 60 ppd to match this resolution limit. Conversely, 20/10 vision would correspond to 120 ppd, and so on. This relationship can be expressed as:

$$\mathrm{ppd} = 60 \cdot r_{\mathrm{snellen}}. \tag{F}$$

Similarly, the mathematical relationship between logMAR and ppd can be expressed by substituting from Eq. (D):

$$\mathrm{ppd} = \frac{60}{10^{\log \mathrm{MAR}}} \tag{G}$$

3 Methods

Further details of the methodology that were not covered in detail in the main text are provided here, including additional figures.

3.1 Apparatus

We used a moving display apparatus as described in Section *Methods - Moving display apparatus* in the main text. Supplementary Figure 1 shows the photograph of the actual setup. Supplementary Figure 2 illustrates the relationship between viewing distance and the effective resolution (ppd) that can be achieved with the moving apparatus. The graph shows how the display's native resolution changes as a function of viewing distance and the subsampled resolutions (×2, ×3, and ×4 subsampling). The advantage of this setup is that we are able to simulate an effectively continuous range of pixel-per-degree (ppd) values for our experiment. We can also simulate the same ppd values at different viewing distances by changing the subsampling resolution. This setup allows for a flexible and precise adjustment of the display's resolution.

The display had a 10-bit colour depth and was calibrated using the Gain-Offset-Gamma (GOG) model to ensure accurate stimulus presentation. Colour measurement patches were displayed using Psychtoolbox running in MATLAB, and their spectra, along with XYZ tristimulus values, were measured using the JETI Specbos 1211 broadband spectroradiometer. This device has a luminance measurement range of 0.2 to 150,000 cd/m^2. All measurements were conducted in a dark room to minimise external light interference. The measurements were used to fit GOG parameters to linearise the display response. The fitted parameters were the black level values and gamma correction for the R, G, and B channels, and the 3x3 transformation matrix between XYZ and linearised RGB colourspace.

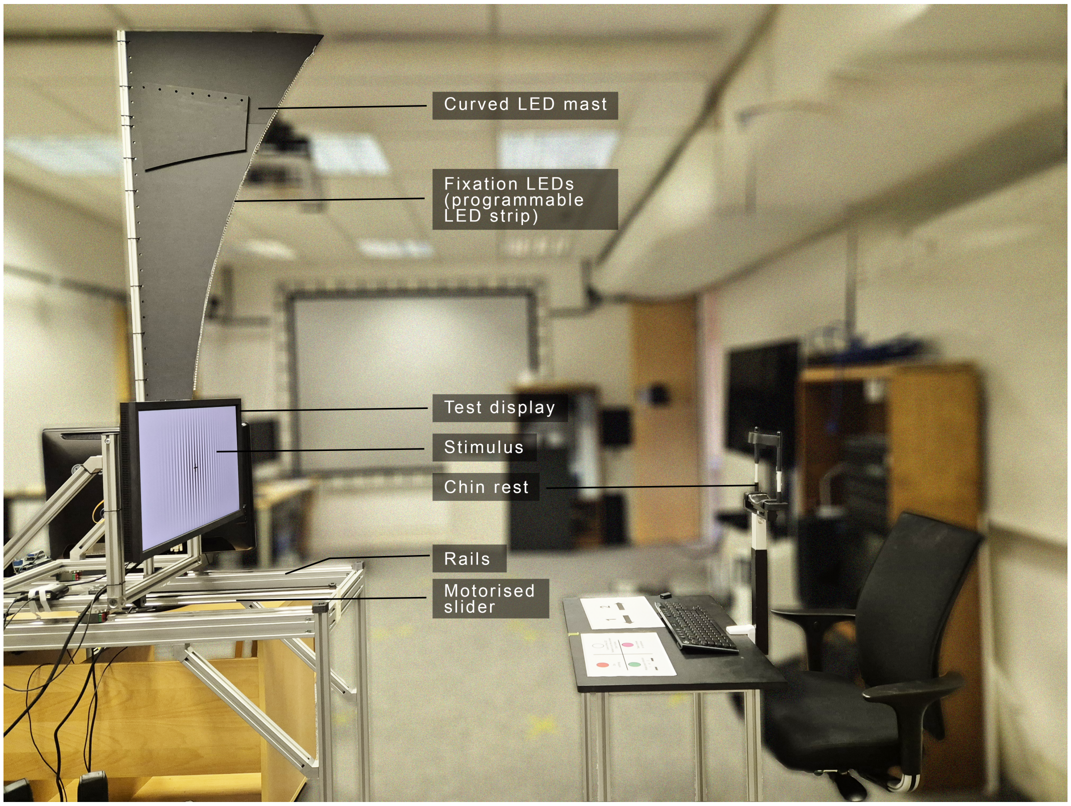

Supplementary Figure 1. Experimental setup. The display can slide on the rails towards and away from the observer. The movement is controlled by a motorized camera slider to show stimulus at different pixel-per-degree (ppd) resolutions. The fixation point for the foveal presentation is the black cross in the centre of the screen. For peripheral viewing, an LED on the curved LED mast is lit up for the corresponding retinal eccentricity.

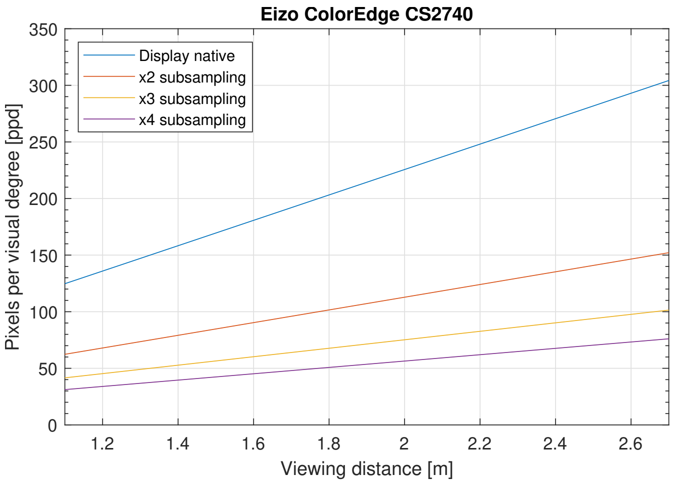

Supplementary Figure 2. The resolution that Eizo ColorEdge CS2740 displays can reproduce at a range of viewing distances.

3.2 Stimuli

As described in Section *Methods - Stimuli*, we used square-wave gratings and text stimuli for our experiments. Supplementary Figure 3 shows a cross-section of an achromatic grating stimulus and the text images. Supplementary Table 1 details the colour coordinates of the gratings used in our experiments. The units of contrast sensitivity and the cone contrast are the same as the ones used in Ashraf et al. (2024)[6].

Supplementary Table 1. Stimuli colour information

Contrast sensitivity	Cone contrast	Colour	Luminance (cd/m^2)	x	y
		Achromatic			
1.09	0.913	White	191	0.3127	0.329
		Black	8.67	0.3127	0.329
		Red-green			
7.42	0.135	Red	100	0.4022	0.2834
		Green	100	0.2410	0.3710
		Yellow-violet			
2.05	0.487	Violet	100	0.2756	0.2394
		Yellow	100	0.3901	0.5157

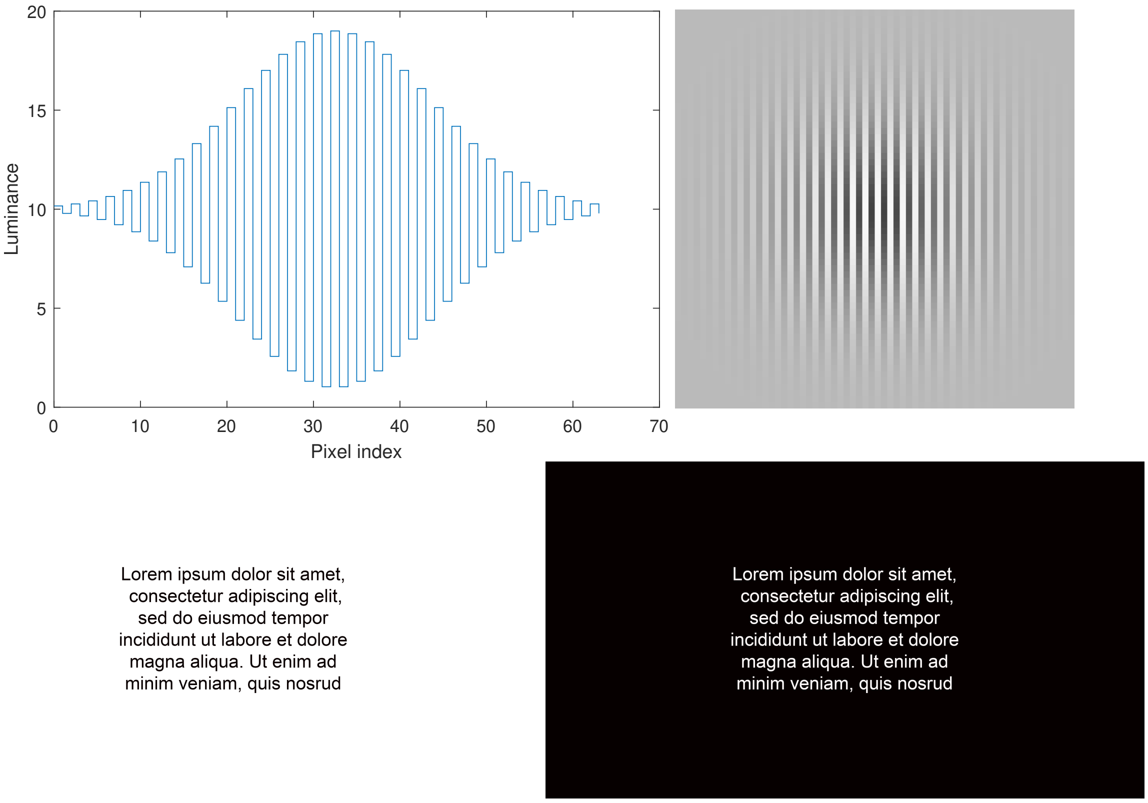

Supplementary Figure 3. a. A cross-section of the square-wave grating, modulated by a Gaussian envelope, generated at the Nyquist frequency. b. the image of the stimulus. c, d. Stimuli for text experiment.

The stimuli were shown at different pixel-per-degree values and the observers' response to whether they could detect the stimulus at the corresponding ppd values were recorded. To modulate the resolution of the stimuli we used the following two strategies:

- Move the display towards or away from the observer using the mechanised camera slider (see Supplementary Figure 1);

- Upsample or downsample the spatial resolution of the screen using integer factors (2×, 3×, 4×,...). The corresponding spatial resolutions are shown as 2×, 3×, and 4× subsampling lines in Supplementary Figure 2.

For each trial, we calculated the viewing distance and the resolution sub-sampling factor that would require the least amount of movement with respect to the display's current position. For example, if the display is currently at a viewing distance of 140 cm and we want to display a stimulus at 50 ppd for our next trial, some of our options within the range of motion of the display are to move the display: i) 51 cm towards the observer at 2x subsampling, ii) 7 cm towards the observer at 3x subsampling, and iii) 38 cm away from the observer at 4x subsampling. The second option in this case requires the display to move only 7 cm and would thus reduce the time required to physically move the display between consecutive trials.

3.3 Prior psychometric function estimation

In order to use an adaptive procedure (QUEST[7]), it is necessary to find a psychometric function whose shape remains the same regardless of the tested ppd value. We used a contrast sensitivity function (CSF) model, castleCSF[6], to estimate the likely psychometric functions for spatial frequency detection thresholds in a 2IFC experiment. The psychometric function used in the CSF model is a Weibull function w.r.t cone contrast value of the stimulus. For different spatial frequencies, the function is displaced along the contrast axis, but the slope of the function does not change. We generated a matrix of these psychometric functions for a dense range of spatial frequencies. Slicing this matrix for our required stimulus contrast effectively produces the psychometric functions w.r.t spatial frequency as shown in Supplementary Figure 4(a). The main difficulty was to find the suitable function of spatial frequency for which the shape of the psychometric function remains consistent for different conditions. Initial attempts to represent spatial frequency on linear, logarithmic, and inverse scales (Supplementary Figure 4(a-c) respectively) revealed varying psychometric function slopes, indicating that the typical assumption of constant slope for psychometric functions in contrast sensitivity experiments may not hold in experiments where spatial frequency is the parameter being measured. To find the optimum scaling, we ran an optimisation routine to minimise the differences in slopes with the exponent of the spatial frequency transformation as a fitted parameter. The value of this fitted parameter was close to 0.3, therefore, a transformation involving the cube root of spatial frequency was explored, which, based on the collected data, provided a more consistent psychometric function across different eccentricities and colour directions. We also used this transformation for scaling the threshold ppd values when fitting models and normalised probability distribution functions:

$$f(\rho) = \sqrt[3]{\rho}, \tag{H}$$

where ρ is the spatial frequency in cpd. The ppd values are twice the cpd values. The resulting psychometric function can be seen in Supplementary Figure 4(d).

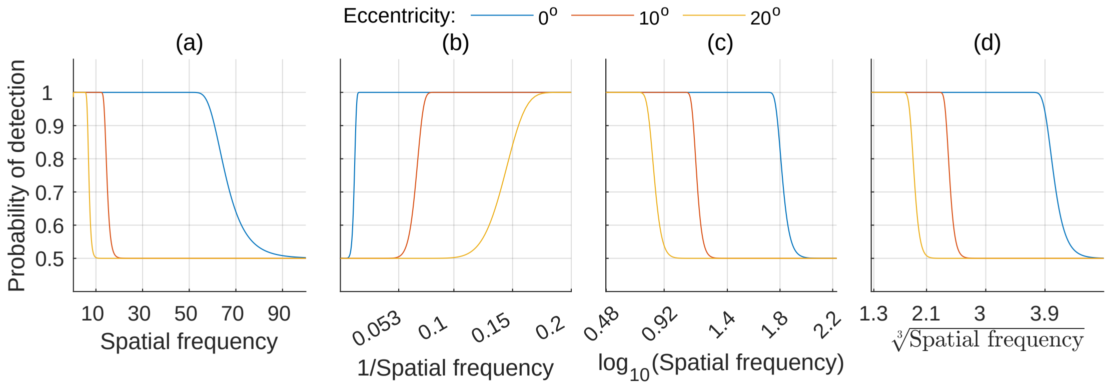

Supplementary Figure 4. Psychometric function used to estimate spatial frequency detection thresholds across different eccentricities (0°, 10°, and 20°). The curves show the probability of detection as functions of spatial frequency, plotted using various transformations.

3.4 Psychophysical method

The study employed a 2-Interval Forced Choice (2IFC) paradigm to measure the minimum resolution required to detect the stimulus. This psychophysical method is advantageous for its sensitivity and accuracy in measuring perceptual thresholds, as it minimises bias by forcing a choice between two presented intervals, one containing the test stimulus described in the section *Stimuli* and the other a uniform field. Participants were seated comfortably in a dark room, facing the display screen at an initial distance of 190 cm. Each trial began with the presentation of a masker for 400 ms, followed by two intervals. The order of

the stimulus and uniform field intervals were randomised across trials to prevent anticipation. Participants were instructed to indicate which interval contained the stimulus and their response was recorded as binary data, representing correct or incorrect decisions. The observers received feedback immediately after their choice.

Each stimulus was presented for 500 ms. The stimulus's onset and exit were modulated by a Gaussian function with $\sigma = 200$ ms. This was to ensure that higher temporal frequencies associated with the onset and exit did not facilitate the detection/discrimination. A sound was played at the onset of each stimulus. QUEST adaptive procedure was used to select the next pixel per degree resolution to be tested, based on the participant's responses. We collected data from 30 to 50 QUEST trials for each condition and each observer. During the display movement, a random noise pattern was shown on the display. The main experiment was completed in three blocks, one for each of the retinal positions that we tested: i) foveal (0°), ii) parafoveal 10°, and iii) parafoveal 20°. The order of the tested colour directions within a session was randomised.

3.5 Data analysis

The individual observers' thresholds are provided in Supplementary Figure 5. The outliers are the individual observations that deviate significantly from the group data, because of individual errors. These deviations can occur due to various factors, including individual variations in visual acuity, attentional lapses, or misunderstanding of task instructions. Particularly in peripheral viewing, where acuity drops markedly, small deviations in gaze direction—a common occurrence given the challenge of maintaining a steady eye position—can result in data points that lie far outside the typical response range. To ensure the robustness of our findings, these outliers were systematically excluded to prevent skewed estimates of the overall psychophysical trends. For each combination of colour direction and eccentricity condition, the median absolute deviation (MAD)[8] of the data from all individual observers was computed. The modified Z-score was then assigned to each data point based on how many MADs away from the median it was. The data points with a modified Z-score greater than 3.5 were considered outliers and systematically removed from the dataset. In total, 13 out of 162 data points were identified as outliers and were excluded from subsequent analyses.

4 Results and discussion

In this section, we provide additional details and data that complement the findings discussed in the main results section of our study. The supplementary materials include extended data tables and figures that show more details about the variability observed across different participants and conditions and more details on the parameters of the model.

4.1 Individual observer data

The raw 2IFC results were fitted with a maximum likelihood function (as described in the *Methods - Data Analysis* section in the main text) for each observer to estimate their individual spatial frequency threshold. Supplementary Figure 5 shows

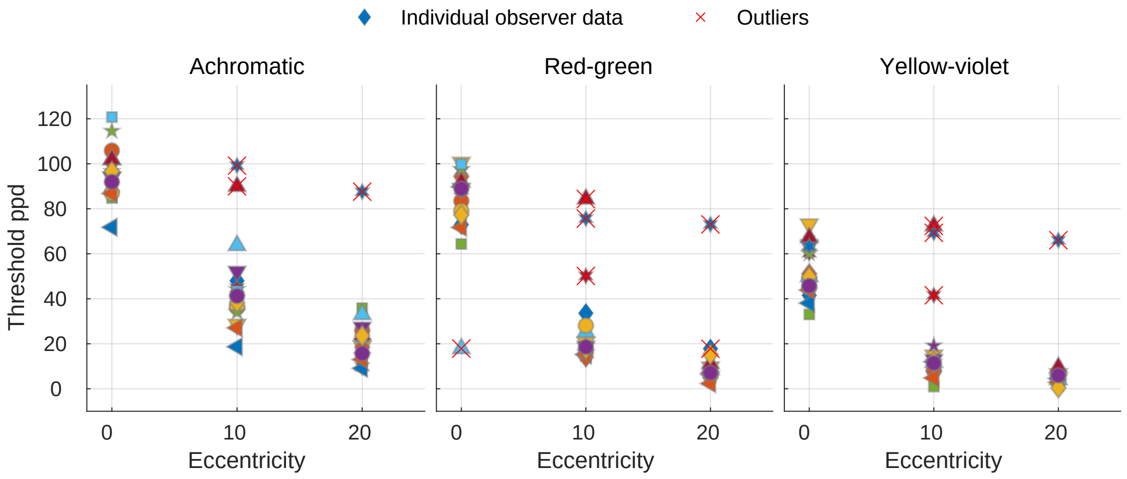

Supplementary Figure 5. Threshold ppd (pixels per degree) values from individual observers across varying retinal eccentricity (in visual degrees) for three colour channels: achromatic, red-green, and yellow-violet. Outliers, indicated by red crosses, were identified and excluded from subsequent analysis. Each colour channel demonstrates a trend of decreasing threshold with increasing eccentricity, reflecting a decline in spatial sensitivity away from the foveal centre.

the threshold ppd values from individual observers across varying retinal eccentricities for three colour channels: achromatic, red-green, and yellow-violet. Outliers, identified by red crosses, were excluded from subsequent analysis to ensure the robustness of the results. Supplementary Table 2 shows the corresponding central tendency and variability of spatial frequency thresholds in pixels per degree (ppd) for the three colour channels at three levels of visual eccentricity (0°, 10°, 20°). The 'Mean' and 'Median' values across all observers, along with their respective 95% Confidence Intervals (CIs), are reported. Additionally, the '95th percentile' values provide the range of threshold values for 95% of the observers. In both the individual and aggregated data, each colour channel demonstrates a trend of decreasing threshold with increasing eccentricity, reflecting a decline in spatial sensitivity away from the foveal centre. This decline reflects the impact of both optical and neural factors on spatial resolution across different regions of the retina.

Supplementary Table 2. The central tendency and variability of spatial frequency thresholds, in pixels per degree (ppd), for different colour channels (achromatic, red-green, yellow-violet) at three levels of visual eccentricity (0, 10, 20 visual degrees). The 'Mean' and 'Median' values across all observers along with the respective 95% Confidence Intervals (CIs) are reported. The '95th percentile' values provide the range of threshold values for 95% of the observers. The data shows a decrease in threshold with increasing eccentricity for each colour direction, indicating a consistent decline in visual sensitivity as the distance from the foveal centre increases.

Color direction	Eccentricity	Mean	[95% CI]	Median	[95% CI]	[95th percentile]
Achromatic	0	95.37	90.45 , 100.7	94.01	87.78 , 100.2	76.65 , 117.2
	10	40.15	35.19 , 45.21	41.40	35.33 , 44.39	22.04 , 58.93
	20	20.81	17.28 , 24.51	20.93	15.69 , 24.18	9.796 , 34.06
Red-green	0	86.77	81.72 , 91.44	89.00	79.05 , 94.21	67.30 , 99.97
	10	19.24	16.82 , 22.09	17.74	15.95 , 18.82	14.32 , 31.12
	20	7.461	6.157 , 8.924	7.146	5.966 , 8.433	3.298 , 13.51
Yellow-violet	0	53.56	48.45 , 58.62	50.49	45.36 , 63.40	35.38 , 70.80
	10	10.55	8.249 , 12.64	11.80	8.038 , 12.05	2.643 , 17.40
	20	4.715	3.660 , 5.800	4.399	3.530 , 5.946	1.109 , 8.703

4.2 Relationship with visual acuity

Visual acuities for the observers in our experiment were estimated using customised Snellen charts shown in Supplementary Figure 6. We used six different Snellen charts, corresponding to the different colour modulations: black-white, red-green, and yellow-violet. The charts were displayed on the same screen used in the main experiment, positioned at a fixed distance of 2.7 m from the observer. The order of the charts' presentation was randomised for each observer. For each chart, observers were asked to read the letters on randomly chosen rows on the chart. Rather than using the traditional line-by-line evaluation, where the observer must correctly identify all letters on a given line, we applied the more sensitive letter-by-letter scoring method

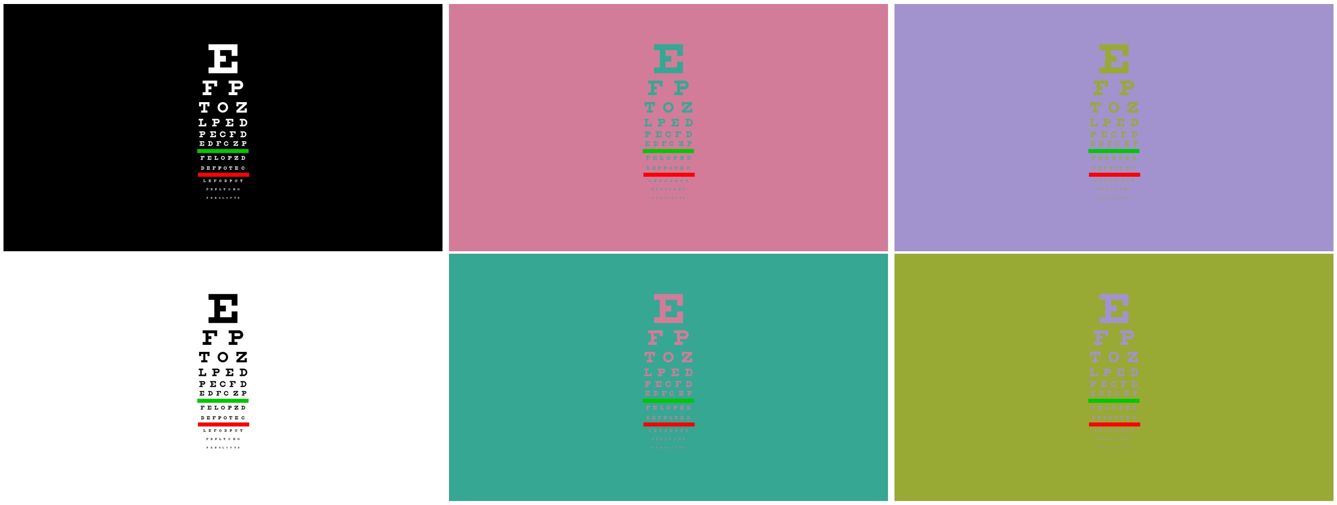

Supplementary Figure 6. Custom Snellen visual acuity charts used in the experiment.

as described in Monaco, Heimerl & Kalb (2009)[9]. This method assigns partial credit based on the proportion of correctly identified letters within each line, which increases the precision of visual acuity measurement. The calculated Snellen fractions were converted to logMAR visual acuity values.

For each colour channel (achromatic, red-green, yellow-violet), visual acuity measurements were averaged across the two opposite polarities. For instance, the achromatic visual acuity was calculated as the average of the measurements from both black-on-white and white-on-black Snellen charts. We then compared these averaged visual acuity values, expressed in logMAR, with the corresponding threshold ppd values derived from our experiment as shown in Supplementary Figure 7. The relationship between these measures was assessed using two-tailed Spearman's rank correlation, with the correlation coefficient (r) and the associated p-value displayed for each colour modulation.

The results indicate that there is no statistically significant correlation between visual acuity and ppd thresholds for the achromatic condition, whereas statistically significant correlations were found for both isoluminant stimuli: red-green and yellow-violet. The low correlation in the achromatic condition may stem from the observers' familiarity with reading black-and-white text, which involves higher-level cognitive processes beyond visual resolution. This suggests that observers may use additional cues for identifying achromatic letters, making the achromatic letter identification task less dependent on low-level visual processing. On the other hand, the significant correlations observed for the isoluminant stimuli indicate that for purely colour-based contrasts, the resolution is more likely to be dictated by low-level visual processes. In these conditions, the visibility of letters appears to be closely tied to the actual resolution of the visual system, as there are fewer additional cues available to the observer.

This finding could suggest the need for future investigations into whether visual acuities measured using letters accurately isolate low-level visual functions or if they are significantly influenced by higher-level contextual cues, such as familiarity with letter shapes and reading habits, and could be used to develop more precise methods of assessing visual acuity that reflect the fundamental capabilities of the visual system.

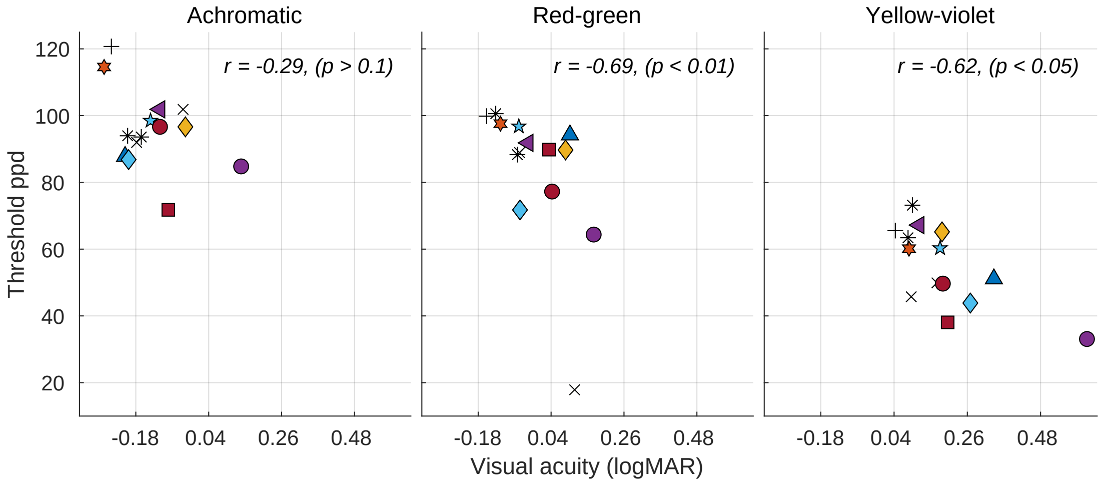

Supplementary Figure 7. Scatter plots showing the relationship between threshold pixels per degree (ppd) and visual acuity (logMAR) for individual observers. Spearman's rank correlation coefficient r and the significance of the correlation are displayed on each plot.

4.3 Parameters of the resolution limit model

The parameters of the model (Eq. 2 in the main paper) were fitted using non-linear regression (`fitnlm` in MATLAB). The model was fitted separately for the three colour directions using the initial parameters reported in Watson (2018)[10]. The fitted curve is shown as dashed lines in Fig. 2 in the main paper. The values of the estimated parameters along with the standard errors and 95% confidence intervals are reported in Supplementary Table 3. Two-tailed t-tests on parameter estimates indicated that all parameters were significantly different from zero ($p < 0.001$). For the assessment of the residuals' normality in our nonlinear model fitting, the Kolmogorov-Smirnov test was employed. For all three colour directions, the test did not reject the null hypothesis of normality with high p-values for achromatic ($p = 0.9456$), red-green ($p = 0.9046$), and yellow-violet ($p = 0.5977$) stimuli, suggesting that the residuals are normally distributed. Supplementary Figure 8 visualises the resulting contrast sensitivity function (CSF) for different colour channels.

Supplementary Table 3. The estimated parameters of the fits for Watson (2018)[10] model. Parameters were estimated using non-linear regression (`fitnlm` in MATLAB) and are reported with 95% confidence intervals, standard errors, t-statistics, and p-values. Separate fits were obtained for each of the three chromatic axes. Parameters were estimated using non-linear regression (`fitnlm` in MATLAB) and are reported with 95% confidence intervals, standard errors, t-statistics, and p-values. Separate fits were obtained for each of the three chromatic axes. Two-tailed t-tests were used to test whether each parameter estimate differed significantly from zero; all parameters were significant with $p < 0.001$. Normality of residuals was confirmed with the Kolmogorov–Smirnov test (Ach: $p = 0.946$, RG: $p = 0.905$, YV: $p = 0.598$). No adjustment for multiple comparisons was applied.

Parameter	Estimate	95% Confidence Interval	Standard Error	t	p-value
		Achromatic			
		Degrees of freedom for error (DFE) = 48			
$\log(S_0^{\mathrm{Ach}})$	2.135	[2.135, 2.135]	4.004e-05	5.331e+04	< 0.001
k_ρ^{Ach}	-0.04394	[-0.04778, -0.0401]	0.00191	-23.01	< 0.001
k_e^{Ach}	0.1601	[0.1294, 0.1908]	0.01527	10.48	< 0.001
		Red-green			
		Degrees of freedom for error (DFE) = 46			
$\log(S_0^{\mathrm{RG}})$	2.179	[2.179, 2.179]	2.609e-05	8.352e+04	< 0.001
k_ρ^{RG}	-0.02997	[-0.03226, -0.02767]	0.00114	-26.28	< 0.001
k_e^{RG}	0.4536	[0.3823, 0.5248]	0.0354	12.81	< 0.001
		Yellow-violet			
		Degrees of freedom for error (DFE) = 46			
$\log(S_0^{\mathrm{YV}})$	1.777	[1.776, 1.777]	0.0001222	1.454e+04	< 0.001
k_ρ^{YV}	-0.05504	[-0.06158, -0.04849]	0.003252	-16.92	< 0.001
k_e^{YV}	0.4630	[0.3462, 0.5798]	0.05804	7.977	< 0.001

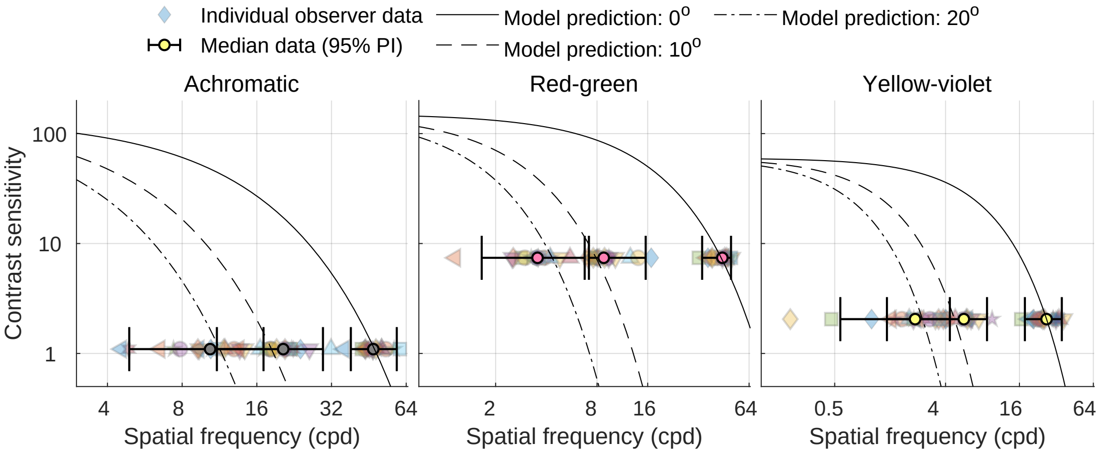

Supplementary Figure 8. Contrast sensitivity functions fitted to the data using the Watson (2018)[10] model. The model fits the parameters of the resulting contrast sensitivity function as functions of spatial frequency and eccentricity. In our experiment, the measured variable was the spatial frequency (in pixels per degree (ppd)) rather than the contrast (unlike conventional contrast sensitivity measurements) and the data points show the measured spatial frequency thresholds for fixed values of contrast. For an ideal display with no gamut limitations, the spatial frequency thresholds would be the values at sensitivity = 1.

4.4 Probability distribution across the population

We model the probability distribution of spatial frequency thresholds (in pixels per degree, ppd) at various retinal eccentricities as normal distributions. These distributions are characterised by parameters, μ_e (mean), σ_e (standard deviation), and A_e (scale). The random variable for these Gaussian distributions is a function of the spatial frequency, $f(\rho)$ as shown in Eq. (H). The value of the scale parameter A_e is fitted such that the integral of the Gaussian distribution, across the transformed spatial frequency $f(\rho)$, equals 1. The central value of the Gaussian (μ_e) distribution at any given eccentricity is a function of the prediction from the model in (Eq. 2 in the main paper).

$$G(\rho;\mu_e,\sigma_e,A_e) = \frac{1}{A_e}\frac{1}{\sigma_e\sqrt{2\pi}}\exp\left(-\frac{(f(\rho)-\mu_e)^2}{2\sigma_e^2}\right). \tag{I}$$

The values of the parameters, σ_e and A_e, at the measured retinal eccentricities, are given in Supplementary Table 4. For eccentricities beyond our measured data points, we employ linear interpolation within the range of measured eccentricities, and linear extrapolation beyond the measured data points, to estimate these parameters. Specifically, we interpolate the standard deviation and scale using MATLAB's `interp1` function with the 'linear' and 'extrap' options.

Supplementary Table 4. The parameters σ_e and A_e for different colour channels (achromatic, red-green, yellow-violet) at three levels of visual eccentricity (0, 10, 20 visual degrees).

Color direction	Eccentricity	σ_e	A_e
Achromatic	0	0.1309	127.3
	10	0.2203	176.0
	20	0.2231	207.2
Red-green	0	0.1161	131.0
	10	0.1452	232.7
	20	0.1809	286.2
Yellow-violet	0	0.1729	154.9
	10	0.2447	280.9
	20	0.2069	343.6

4.5 The effect of viewing distance

We control the resolution of the stimulus in our experiment by adjusting the viewing distance. Yet, the viewing distance may have a sizeable effect on the detection of high-frequency patterns[11–13], with perceived resolution expected to be higher at larger distances. This is due to optical factors: diffraction and accommodative error. As the stimulus gets closer to our eyes, the pupil contracts (miosis). While the smaller pupil restricts the amount of light entering the eye and makes diffraction stronger, it also reduces optical aberrations, and improves depth-of-field at smaller distances, increasing the maximum perceivable resolution. Conversely, larger viewing distances reduce accommodation errors[14] and, therefore, retinal blur, enhancing the ability to perceive high frequencies.

We tested the effect of viewing distance in an additional experiment involving 16 observers. Stimuli with the same pixel-per-degree resolution were displayed at two different viewing distances, termed as *far* and *near*. The experiment was conducted only for the foveal condition, after the main experiment, to specifically test the effect of viewing distance on detection thresholds. Supplementary Figure 9 shows the probabilities of detection for stimuli shown at two viewing distances for different observers (estimated from 20 measurements). The viewing distances for each observer were chosen according to their threshold ppd values for the corresponding colour direction. To test the statistical significance of differences between the two viewing distances, we calculated the probabilities of difference between the two binomial distributions for *near* and *far* responses. To account for multiple comparisons, we applied the Holm-Bonferroni correction to the p-values from the two-tailed tests (at $\alpha = 0.05$). After correction, we found that the differences in probabilities were statistically significant only for 2 observers and for the yellow-violet stimuli (see the circled data points in Supplementary Figure 9).

To estimate the difference in ppd thresholds, we predicted the equivalent ppd thresholds for the *near* and *far* data from their probability of detection values using our psychometric function. Supplementary Figure 10 shows the differences in predicted thresholds between the two viewing distances. Negative values indicate higher thresholds for the *near* conditions, and vice versa. For most observers, the differences between the two conditions were small within the range of our tested distances. The 2 yellow-violet differences identified as statistically significant are highlighted with red circles in Supplementary Figure 10. Our data indicates that the effect of viewing distance on the resolution limit is small and inconsistent across the observers. Our

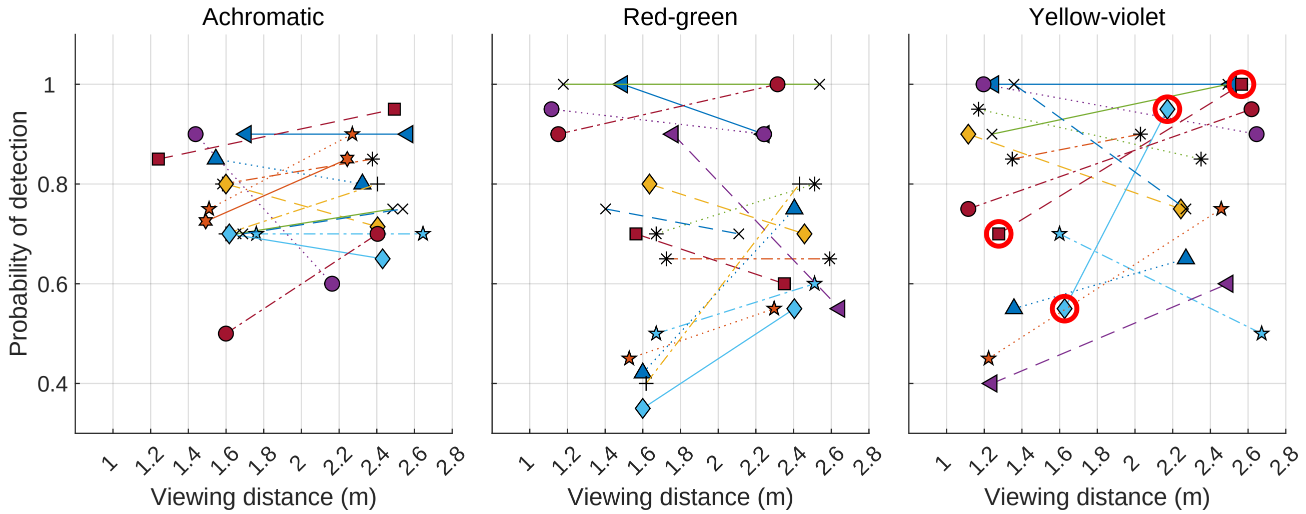

Supplementary Figure 9. Difference in the probability of detection between *far* and *near* stimuli. The figure shows individual observer data for achromatic, red-green, and yellow-violet stimuli at different viewing distances (m) for 16 observers. Each symbol represents data from a single observer. Negative slopes indicate higher detection probabilities for the near condition, while positive slopes indicate higher detection probabilities for the far condition. The data illustrate the variability in detection probability with changes in viewing distance, highlighting individual differences in visual sensitivity and the impact of viewing distance on the detection of spatial frequency across different colour channels. The data points with statistically significant differences between the two viewing conditions are highlighted with red circles.

protocol did not allow us to confirm statistical significance for the majority of the observers. For that reason, we do not account for the effect of viewing distance when reporting the results of our main experiment.

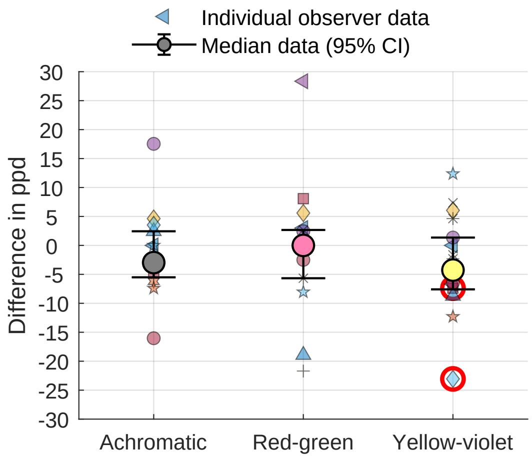

Supplementary Figure 10. Difference in ppd thresholds between *far* and *near* stimuli. The figure shows individual observer data and median data with 95% prediction intervals for achromatic, red-green, and yellow-violet stimuli for 16 observers. Negative values indicate higher thresholds for the near condition, while positive values indicate higher thresholds for the far condition. The red circles highlight individual data points, where the difference in binomial distributions of observer responses was found to be significant.

4.6 Foveated filtering

Our foveated rendering application in Section *Example: Foveated rendering* shows an example of eccentricity-dependent filtering on an image. The high-frequency details and the sub-threshold contrasts are selectively removed, based on retinal eccentricity, according to the predictions of our model. Supplementary Figure 11 presents the decomposition of the original and

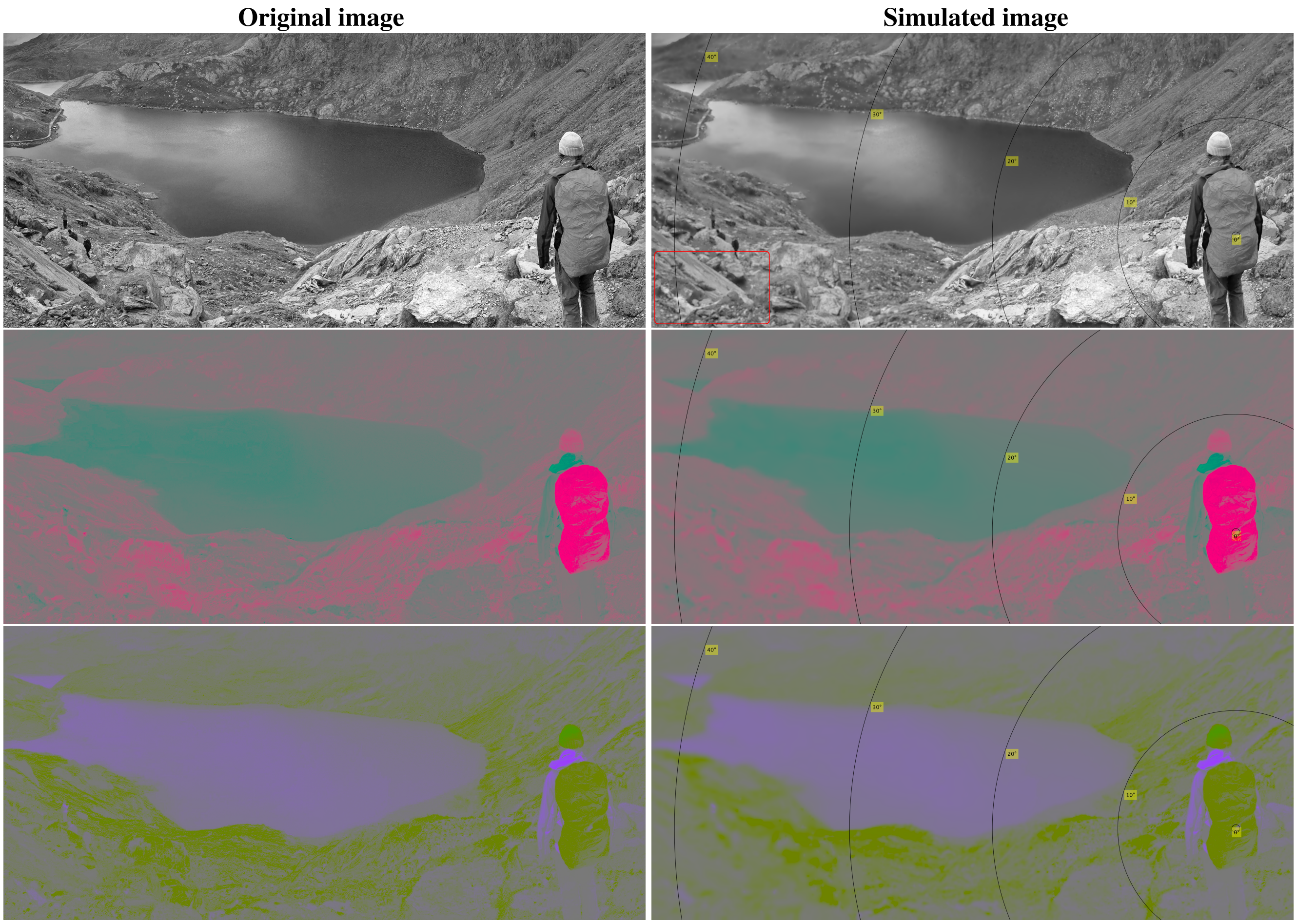

Supplementary Figure 11. Comparison of original and simulated images decomposed into three colour planes. The figure illustrates how foveated filtering selectively reduces spatial details based on retinal eccentricity, particularly in chromatic planes (red-green and yellow-violet). The simulated images (right) shows diminished detail in peripheral areas, consistent with the filtering thresholds applied in the rendering process. To observe the intended effect, zoom into the figure such that the red outline in the top right image is approximately the size of a standard credit card.

processed images into their respective colour planes (achromatic, red-green, and yellow-violet). The original images on the left maintain full resolution across all colour channels, while the simulated images on the right show a progressive loss of detail, particularly in the red-green and yellow-violet planes.

Supplementary Figure 12 dissects the image processing by displaying the individual spatial frequency bands obtained from the Laplacian pyramid decomposition. Each row corresponds to a different frequency band, ranging from low to high spatial frequencies, filtered with an eccentricity-dependent mask. The progressive removal of high-frequency details, especially at larger eccentricities, is evident, particularly in the higher frequency bands (2.83 cpd and above).

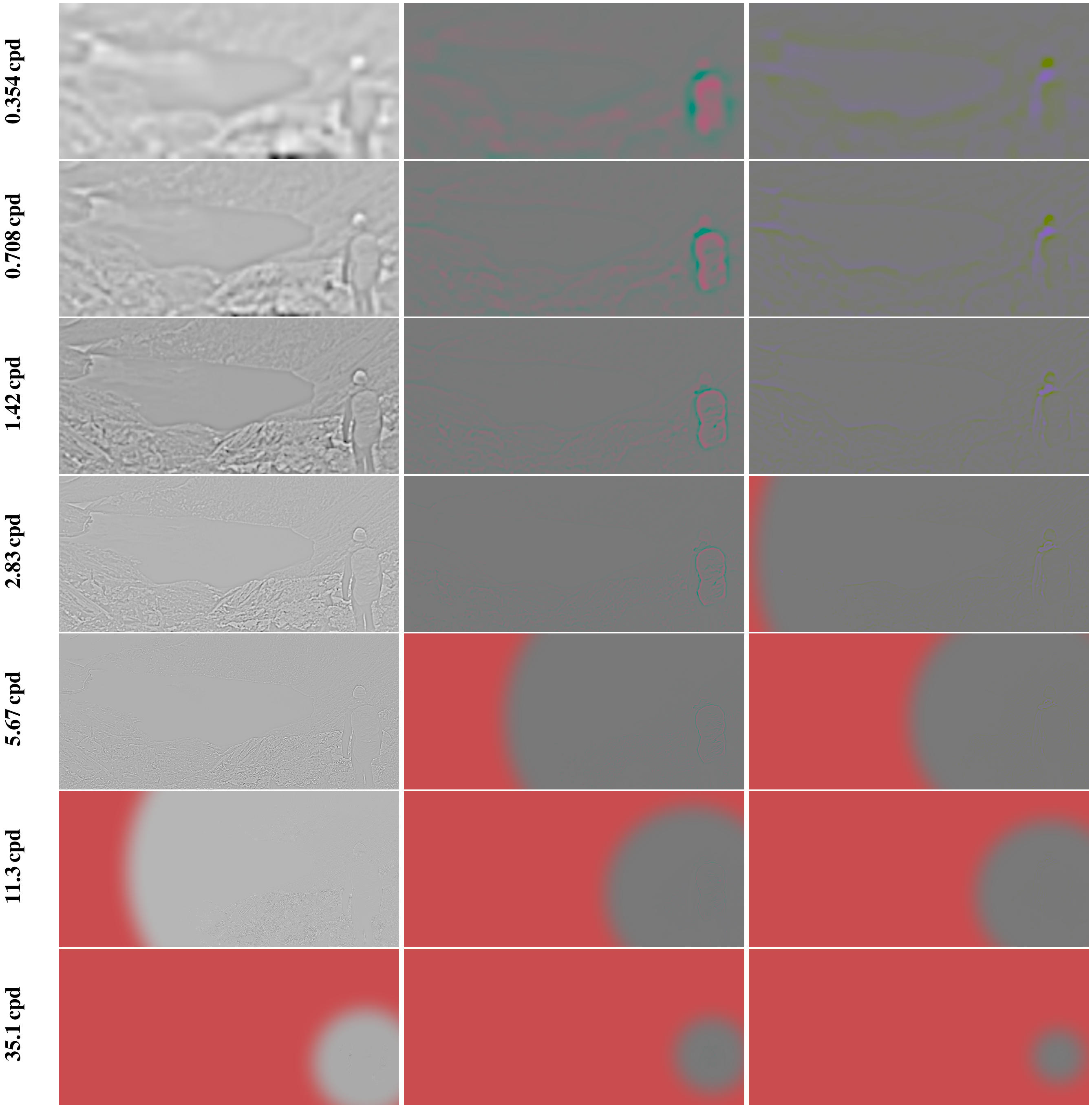

Supplementary Figure 12. Spatial frequency decomposition of the colour planes using a Laplacian pyramid coupled with the filtering mask. Each row corresponds to a different frequency band. The high-frequency details are progressively attenuated in the filtered image as a function of retinal eccentricity, preserving essential visual information while removing imperceptible details in the periphery. The red portions of the image show the areas where all the contrast information is removed.

References

1. Velasco e Cruz, A. A. Historical roots of 20/20 as a (wrong) standard value of normal visual acuity. *Optom. vision science* **67**, DOI: 10.1097/00006324-199008000-00022 (1990).

2. Helmholtz, H. v. & Nagel, W. Minimum dimensions of perceptible objects. In *Treatise on Physiological Optics*, vol. 3 (Dover, 1962).

3. Snellen, H. On the methods of determining the acuity of vision. *Syst. diseases eye* **2**, 93–98 (1897).

4. Snyder, C. Herman Snellen and V= d/D. *Arch. Ophthalmol.* **68**, 571–573, DOI: 10.1001/archopht.1962.00960030575025 (1962).

5. Mantiuk, R. K. *et al.* FovVideoVDP: A visible difference predictor for wide field-of-view video. *ACM Transaction on Graph.* **40**, 49, DOI: 10.1145/3450626.3459831 (2021).

6. Ashraf, M., Mantiuk, R. K., Chapiro, A. & Wuerger, S. castleCSF—A contrast sensitivity function of color, area, spatiotemporal frequency, luminance and eccentricity. *Journal of Vision* **24**, 5–5, DOI: 10.1167/jov.24.4.5 (2024).

7. Watson, A. B. & Pelli, D. G. QUEST: A Bayesian adaptive psychometric method. *Percept. & psychophysics* **33**, 113–120, DOI: 10.3758/BF03202828 (1983).

8. Leys, C., Ley, C., Klein, O., Bernard, P. & Licata, L. Detecting outliers: Do not use standard deviation around the mean, use absolute deviation around the median. *J. experimental social psychology* **49**, 764–766, DOI: 10.1016/j.jesp.2013.03.013 (2013).

9. Monaco, W. A., Heimerl, J. M. & Kalb, J. T. *A Clinically Useful Tool to Determine an Effective Snellen Fraction: Details*, vol. 4756 (Army Research Laboratory, 2009).

10. Watson, A. B. The field of view, the field of resolution, and the field of contrast sensitivity. *Electron. Imaging* **30**, 1–11, DOI: 10.2352/J.Percept.Imaging.2018.1.1.010505 (2018).

11. Depalma, J. J. & Lowry, E. M. Sine-Wave Response of the Visual System. II. Sine-Wave and Square-Wave Contrast Sensitivity. *J. Opt. Soc. Am.* **52**, 328, DOI: 10.1364/JOSA.52.000328 (1962).

12. Schober, H. A. W. & Hilz, R. Contrast sensitivity of the human eye for square-wave gratings. *J. Opt. Soc. Am.* **55**, 1086, DOI: 10.1364/JOSA.55.001086 (1965).

13. Hernández, C., Doménech, B., Seguí, M. M. & Illueca, C. The effect of pupil and observation distance on the contrast sensitivity function. *Ophthalmic Physiol. Opt.* **16**, 336–341, DOI: 10.1046/j.1475-1313.1996.95001166.x (1996).

14. Charman, W. N. & Radhakrishnan, H. Accommodation, pupil diameter and myopia. *Ophthalmic Physiol. Opt.* **29**, 72–79, DOI: 10.1111/j.1475-1313.2008.00611.x (2009).
