# Supplementary material for: Resolution limit of the eye — how many pixels can we see?
Source: Nat Commun. 2025 Oct 27;16:9086. doi: 10.1038/s41467-025-64679-2 (PMC12559231; doi:10.1038/s41467-025-64679-2)
Supplement: Supplementary file 2 — Reporting Summary [file 41467_2025_64679_MOESM2_ESM.pdf]

nature portfolio

Corresponding author(s): Rafal Mantiuk

Last updated by author(s): Aug 22, 2025

Reporting Summary

Nature Portfolio wishes to improve the reproducibility of the work that we publish. This form provides structure for consistency and transparency in reporting. For further information on Nature Portfolio policies, see our Editorial Policies and the Editorial Policy Checklist.

Statistics

For all statistical analyses, confirm that the following items are present in the figure legend, table legend, main text, or Methods section.

n/a	Confirmed	
☐	☒	The exact sample size (*n*) for each experimental group/condition, given as a discrete number and unit of measurement
☐	☒	A statement on whether measurements were taken from distinct samples or whether the same sample was measured repeatedly
☐	☒	The statistical test(s) used AND whether they are one- or two-sided<br>*Only common tests should be described solely by name; describe more complex techniques in the Methods section.*
☒	☐	A description of all covariates tested
☐	☒	A description of any assumptions or corrections, such as tests of normality and adjustment for multiple comparisons
☐	☒	A full description of the statistical parameters including central tendency (e.g. means) or other basic estimates (e.g. regression coefficient) AND variation (e.g. standard deviation) or associated estimates of uncertainty (e.g. confidence intervals)
☐	☒	For null hypothesis testing, the test statistic (e.g. F, t, r) with confidence intervals, effect sizes, degrees of freedom and P value noted<br>*Give P values as exact values whenever suitable.*
☒	☐	For Bayesian analysis, information on the choice of priors and Markov chain Monte Carlo settings
☒	☐	For hierarchical and complex designs, identification of the appropriate level for tests and full reporting of outcomes
☐	☒	Estimates of effect sizes (e.g. Cohen's *d*, Pearson's *r*), indicating how they were calculated

Our web collection on statistics for biologists contains articles on many of the points above.

Software and code

Policy information about availability of computer code

Data collection	The experiment was implemented in Matlab 2023b with Psychtoolbox
Data analysis	The data was analyzed with Matlab 2024a and R2022b. The code for reproducing the plots from Fig. 2 is also made available at https://github.com/gfxdisp/resolution_limit and an additional web-based calculator is available at https://www.cl.cam.ac.uk/research/rainbow/projects/display_calc/.

For manuscripts utilizing custom algorithms or software that are central to the research but not yet described in published literature, software must be made available to editors and reviewers. We strongly encourage code deposition in a community repository (e.g. GitHub). See the Nature Portfolio guidelines for submitting code & software for further information.

Data

Policy information about availability of data

All manuscripts must include a data availability statement. This statement should provide the following information, where applicable:

- Accession codes, unique identifiers, or web links for publicly available datasets
- A description of any restrictions on data availability
- For clinical datasets or third party data, please ensure that the statement adheres to our policy

The raw and processed data from our study can be found at https://github.com/gfxdisp/resolution_limit with no restrictions.

Research involving human participants, their data, or biological material

Policy information about studies with human participants or human data. See also policy information about sex, gender (identity/presentation), and sexual orientation and race, ethnicity and racism.

Reporting on sex and gender	We identify the number of male and female participants, which indicates both sex and gender.
Reporting on race, ethnicity, or other socially relevant groupings	Race and ethnicity are not reported.
Population characteristics	We relied on a younger population of observers (the mean age of 25.5, age range: 13-46 years) to ensure healthy visual function.
Recruitment	Participants were recruited via university mailing lists and word-of-mouth, which may introduce self-selection bias (e.g. volunteers with interest in vision studies, healthier vision than average). Most were students or members of the research group. As such, the sample may reflect a relatively narrow demographic in terms of age and educational background. While this is common in laboratory-based studies, it may influence the generalisability of the findings to broader populations.
Ethics oversight	The experiment was approved by the departmental ethical committee at the Department of Computer Science and Technology of the University of Cambridge

Note that full information on the approval of the study protocol must also be provided in the manuscript.

Field-specific reporting

Please select the one below that is the best fit for your research. If you are not sure, read the appropriate sections before making your selection.

☐ Life sciences ☒ Behavioural & social sciences ☐ Ecological, evolutionary & environmental sciences

For a reference copy of the document with all sections, see nature.com/documents/nr-reporting-summary-flat.pdf

Behavioural & social sciences study design

All studies must disclose on these points even when the disclosure is negative.

Study description	This was a quantitative experimental study using psychophysical methods to measure visual spatial resolution limits under different stimulus conditions (colour direction, retinal eccentricity). Data were collected using forced-choice detection tasks.
Research sample	The research sample consisted of 18 adult participants (6 female, 12 male), with a mean age of 25.5 (age range: 13-46 years), recruited from the university community, primarily students and researchers. All participants had normal or corrected-to-normal visual acuity and normal colour vision (assessed using standard clinical tests). While the sample is not representative of the general population, it is typical for controlled laboratory studies in vision science.
Sampling strategy	Participants were recruited using convenience sampling via university mailing lists and word-of-mouth. No formal power analysis was performed; the sample size was based on similar prior studies and was sufficient to identify consistent individual and group-level effects in visual threshold estimation.
Data collection	Data were collected in a controlled laboratory setting using a calibrated monitor for stimulus presentation and a computer-based psychophysical platform (MATLAB with Psychtoolbox) for response recording. Participants performed detection tasks while seated in front of the display. The experimenter was present during parts of the sessions to monitor fixation compliance, but no feedback or verbal interaction occurred during trials. The researcher was not blind to the study conditions, but stimulus presentation was randomised to reduce bias.
Timing	Data collection for the main experiment (involving square-wave gratings) was conducted between 1st and 21st March 2024. The secondary experiment using text stimuli was conducted between 10th and 13th June 2024. Each participant completed all conditions in three one-hour sessions for the main experiment, and in a single one-hour session for the secondary experiment.
Data exclusions	A total of 13 out of 162 data points were excluded from the analysis due to being identified as outliers using a modified Z-score method. These outliers were excluded to prevent skewed estimates of psychophysical trends. All exclusions were performed based on pre-defined criteria, and the excluded data points are shown in Supplementary Figure E.
Non-participation	All participants who consented to participate completed the study. There were no dropouts or excluded participants.
Randomization	Participants were not assigned to experimental groups. All participants completed the same set of tasks, but the order of stimulus conditions was randomised within subjects.

Reporting for specific materials, systems and methods

We require information from authors about some types of materials, experimental systems and methods used in many studies. Here, indicate whether each material, system or method listed is relevant to your study. If you are not sure if a list item applies to your research, read the appropriate section before selecting a response.

Materials & experimental systems

n/a	Involved in the study
☒	☐ Antibodies
☒	☐ Eukaryotic cell lines
☒	☐ Palaeontology and archaeology
☒	☐ Animals and other organisms
☒	☐ Clinical data
☒	☐ Dual use research of concern
☒	☐ Plants

Methods

n/a	Involved in the study
☒	☐ ChIP-seq
☒	☐ Flow cytometry
☒	☐ MRI-based neuroimaging

Plants

Seed stocks	*Report on the source of all seed stocks or other plant material used. If applicable, state the seed stock centre and catalogue number. If plant specimens were collected from the field, describe the collection location, date and sampling procedures.*
Novel plant genotypes	*Describe the methods by which all novel plant genotypes were produced. This includes those generated by transgenic approaches, gene editing, chemical/radiation-based mutagenesis and hybridization. For transgenic lines, describe the transformation method, the number of independent lines analyzed and the generation upon which experiments were performed. For gene-edited lines, describe the editor used, the endogenous sequence targeted for editing, the targeting guide RNA sequence (if applicable) and how the editor was applied.*
Authentication	*Describe any authentication procedures for each seed stock used or novel genotype generated. Describe any experiments used to assess the effect of a mutation and, where applicable, how potential secondary effects (e.g. second site T-DNA insertions, mosiacism, off-target gene editing) were examined.*
